# Supplementary material for: Telencephalic outputs from the medial entorhinal cortex are copied directly to the hippocampus
Source: eLife. 2022 Feb 21;11:e73162. doi: 10.7554/eLife.73162 (PMC8940174; doi:10.7554/eLife.73162)
Supplement: Transparent reporting form [file elife-73162-transrepform1.docx]

***eLife’s* transparent reporting form**

We encourage authors to provide detailed information *within their submission* to facilitate the interpretation and replication of experiments. Authors can upload supporting documentation to indicate the use of appropriate reporting guidelines for health-related research (see [EQUATOR Network](http://www.equator-network.org/%20)), life science research (see the [BioSharing Information Resource](https://biosharing.org/)), or the [ARRIVE guidelines](http://www.plosbiology.org/article/info:doi/10.1371/journal.pbio.1000412) for reporting work involving animal research. Where applicable, authors should refer to any relevant reporting standards documents in this form.

If you have any questions, please consult our Journal Policies and/or contact us: [editorial@elifesciences.org](mailto:editorial@elifesciences.org).

**Sample-size estimation**

- You should state whether an appropriate sample size was computed when the study was being designed
- You should state the statistical method of sample size computation and any required assumptions
- If no explicit power analysis was used, you should describe how you decided what sample (replicate) size (number) to use

Please outline where this information can be found within the submission (e.g., sections or figure legends), or explain why this information doesn’t apply to your submission:

Sample sizes were not predetermined based on statistical methods. This is largely because we do not compare groups, rather we study the organizing principles of the brain that does not vary largely from individual to individual. Therefore, our sample sizes are similar to other studies that investigate similar phenomena.

**Replicates**

- You should report how often each experiment was performed
- You should include a definition of biological versus technical replication
- The data obtained should be provided and sufficient information should be provided to indicate the number of independent biological and/or technical replicates
- If you encountered any outliers, you should describe how these were handled
- Criteria for exclusion/inclusion of data should be clearly stated
- High-throughput sequence data should be uploaded before submission, with a private link for reviewers provided (these are available from both GEO and ArrayExpress)

Please outline where this information can be found within the submission (e.g., sections or figure legends), or explain why this information doesn’t apply to your submission:

All experiments were replicated using the sample sizes described in the manuscript.

In experiments where EC L5a neurons were targeted to investigate axon topography in the hippocampus and in patch-clamp experiments where functional connectivity was probed, brains in which labelled neurons were found outside of EC L5 were excluded. (These were brains with occasional and sparse labelling in L2 or L3 or the parasubiculum.) In addition, in patch-clamp experiments, neurons with a resting membrane potential less negative than -50 mV or a bridge balance higher than 40 MOhm were excluded from the analysis, as were neurons that did not fit within any cell group that we defined using previously published criteria.

For MAPseq, some telencephalic brain areas were excluded from the study because of the difficulty in dissecting or identifying brain areas in the sagittal plane. All sections > 3.7 mm lateral to bregma which are not annotated in Allen Brain Reference Atlas were excluded. Therefore, neocortical areas in the most lateral sections such as perirhinal cortex, ectorhinal cortex, temporal association areas were not included in the study. The claustrum and adjacent neocortical areas (visceral, agranular insular, gustatory) were excluded as it was not possible to separate these areas precisely to prevent contamination between the assigned divisions. Since borders between the brain divisions CNU and OLF/Ctxsp were not always clear, dissections avoided these areas hence the brain areas in these divisions are partially included. White matter between the hippocampus and the neocortex carrying axon tracts were also excluded.

Orphan barcodes, barcodes which did not have counts in the injection site, (dMEC or vMEC) were removed. We then calculated the 95th percentile of the barcode counts in our negative controls and based on this set all barcode counts of 1 to 0. A small number of barcodes had a higher count in any target area compared to the injection site which might be a result of incomplete dissection of the injection site or viral expression in areas that the virus spilled into as we retracted the pipette. These barcodes were removed. Since our goal was to find whether neurons projecting to the telencephalon also project to the hippocampus we removed the barcodes that had no counts in any of the telencephalic target areas. For the same reason, barcodes that were detected only in the dHip or vHip hippocampus were also removed. Finally, we excluded all barcodes with counts of less than 400 in the injection site to minimise the possibility of incomplete transport of RNA barcodes to axons in target areas due to weak expression at the cell bodies or low counts due to PCR or polymerase errors. Barcodes with low counts in all target areas (< 10) were also excluded to account for potential false positives.

**Statistical reporting**

- Statistical analysis methods should be described and justified
- Raw data should be presented in figures whenever informative to do so (typically when N per group is less than 10)
- For each experiment, you should identify the statistical tests used, exact values of N, definitions of center, methods of multiple test correction, and dispersion and precision measures (e.g., mean, median, SD, SEM, confidence intervals; and, for the major substantive results, a measure of effect size (e.g., Pearson's r, Cohen's d)
- Report exact p-values wherever possible alongside the summary statistics and 95% confidence intervals. These should be reported for all key questions and not only when the p-value is less than 0.05.

Please outline where this information can be found within the submission (e.g., sections or figure legends), or explain why this information doesn’t apply to your submission:

Results of all statistical analyses were reported in the main text or figure legends.

(For large datasets, or papers with a very large number of statistical tests, you may upload a single table file with tests, Ns, etc., with reference to sections in the manuscript.)

**Group allocation**

- Indicate how samples were allocated into experimental groups (in the case of clinical studies, please specify allocation to treatment method); if randomization was used, please also state if restricted randomization was applied
- Indicate if masking was used during group allocation, data collection and/or data analysis

Please outline where this information can be found within the submission (e.g., sections or figure legends), or explain why this information doesn’t apply to your submission:

Group allocation does not apply to the study. Sex of the animal was randomly chosen in all experiments.

**Additional data files (“source data”)**

- We encourage you to upload relevant additional data files, such as numerical data that are represented as a graph in a figure, or as a summary table
- Where provided, these should be in the most useful format, and they can be uploaded as “Source data” files linked to a main figure or table
- Include model definition files including the full list of parameters used
- Include code used for data analysis (e.g., R, MatLab)
- Avoid stating that data files are “available upon request”

Please indicate the figures or tables for which source data files have been provided:

We provide the barcode count matrix from MAPseq experiments.
